# A novel biological model for training in percutaneous renal access

**DOI:** 10.1080/2090598X.2019.1642600

**Published:** 2019-08-08

**Authors:** Mohankumar Vijayakumar, Sudharsan Balaji, Abhishek Singh, Arvind Ganpule, Ravindra Sabnis, Mahesh Desai

**Affiliations:** Department of Urology, Muljibhai Patel Urological Hospital, Nadiad, India

**Keywords:** Percutaneous renal access, simulator, puncture, bovine kidney

## Abstract

**Objective**: To develop a new model comprised of a bovine kidney within a chicken carcass for training in percutaneous renal access (PRA) and compare its effectiveness with the traditional mannequin model.

**Subjects, materials and methods**: The study was conducted from January 2017 to June 2017. The content and the construct validity of the new model were confirmed after which it was compared with the traditional non-biological model for PRA. In all, 20 urology residents, with experience of <20 cases, were enrolled in the study. The parameters assessed were time to puncture, attempts to successful puncture, and fluoroscopy exposure time. They were also asked to complete a subjective assessment questionnaire.

**Results**: The new *ex vivo* biological model had both content and construct validity. On comparison with the non-biological model, there was no statistically significant difference between the two models for time to puncture, total fluoroscopy exposure, and also the number of attempts taken for a successful puncture. The participants felt that the new biological model was better than the non-biological model in terms of overall assessment, tissue feel, and confidence in training. But the non-biological model scored better than the new biological model for ease of puncture and model preparation.

**Conclusion**: The present model is inexpensive and easy to construct, and has both content and construct validity. It is a feasible model for fluoroscopy-guided PRA.

**Abbreviations:** 3D: three-dimensional; PCNL: percutaneous nephrolithotomy; PRA: percutaneous renal access; VR: virtual reality

## Introduction

Percutaneous nephrolithotomy (PCNL) is the treatment of choice for complex renal calculi, especially for stones >2 cm. PCNL has come a long way since its introduction in 1976 [1]. The immense advance in technology has led to a reduction in tract size and subsequently complications. Even with such massive technological advances in optics and energy devices, the fundamental and most important aspect in a PCNL, the access to the pelvicalyceal system, remains the same. The puncture is the most important step in PCNL [2,3]. The famous saying ‘Well begun is half done’ holds perfectly true for PCNL. Perfect puncture is imperative in performing PCNL without complications, the learning curve for which is steep [2]. The puncture can be done either by a urologist or a radiologist. Although 69.7% of urologists perform various percutaneous renal procedures, only a few amongst them create their own access [4,5]. There is evidence in the literature that urologists who do their own punctures tend to have better outcomes [4]. Access to the pelvicalyceal system helps not only in PCNL, but also in cases involving antegrade stenting, endopyelotomy, diversion of urine in obstructive uropathy, and percutaneous resection of pelvic tumours.

Studies indicate that the learning curve for good PCNL is steep. It takes a minimum of 20 cases to learn the basic skills, ~60 cases to achieve surgical competence, and ~100 cases to achieve surgical excellence [3,6]. Despite the steep learning curve, there is paucity of cheap and high-fidelity models. The training models available at present are either computer-based virtual-reality (VR) simulators or mannequin based or biological models. There is no data in the literature comparing biological and non-biological models for training in percutaneous renal access (PRA).

In the present study, we aimed to develop a new inexpensive model comprising a bovine kidney within a chicken carcass (biological model) for training in PRA with high fidelity and compare its effectiveness with the traditional mannequin (non-biological) model.

## Subjects, materials and methods

The study was conducted in Muljibhai Patel Urological Hospital, Nadiad Gujarat from January to June 2017. The content and the construct validity of the new biological model were confirmed after which it was compared with the traditional mannequin model for PRA.

### Preparation of the ex vivo model

**Figure 1.**
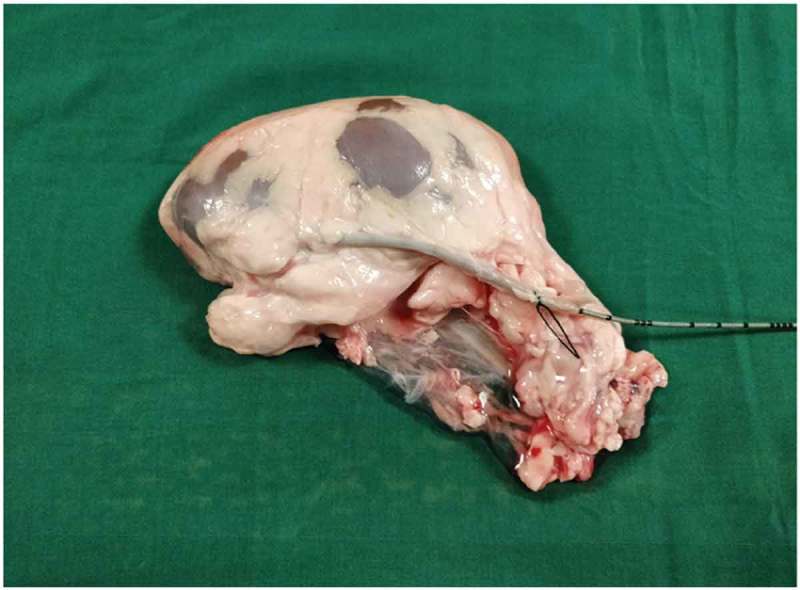
Bovine kidney with ureteric catheter in the ureter.

**Figure 2.**
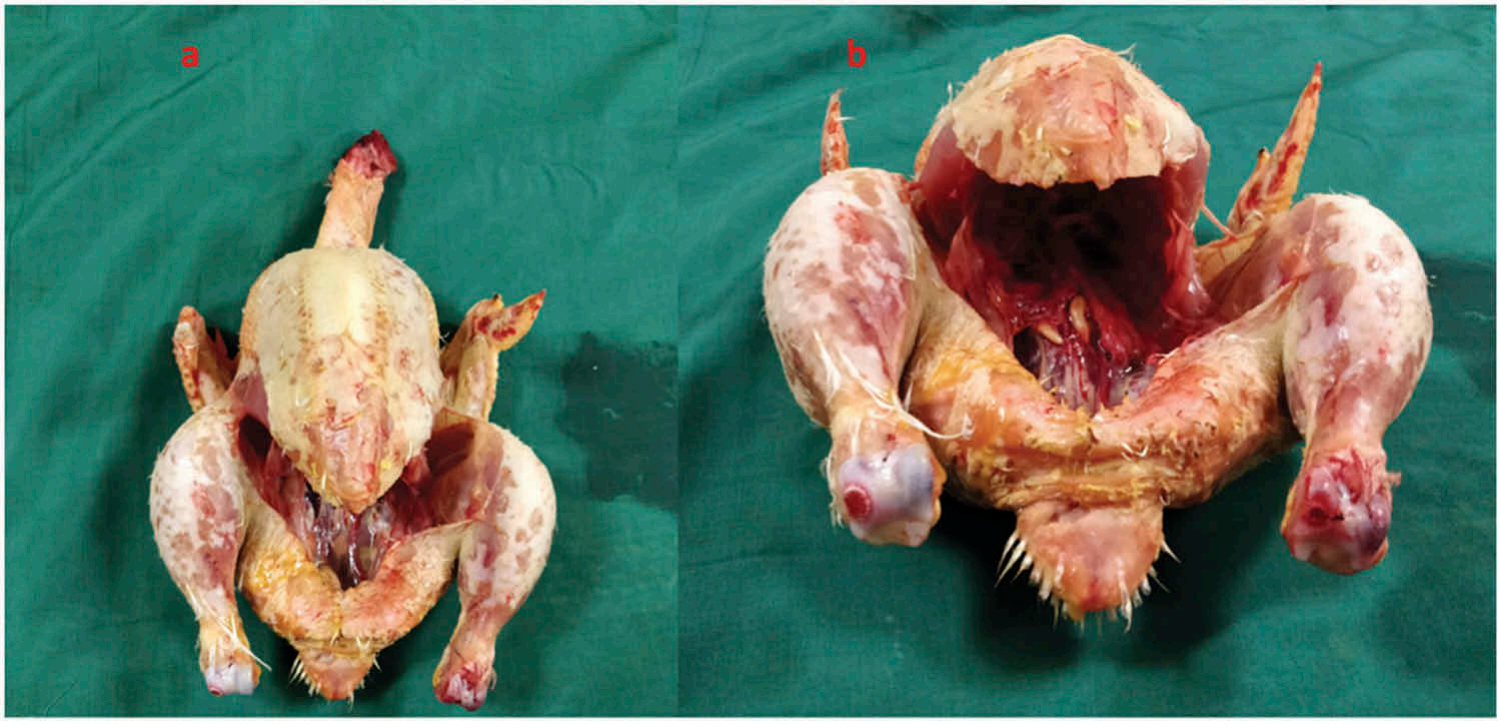
Eviscerated chicken carcass.

**Figure 3.**
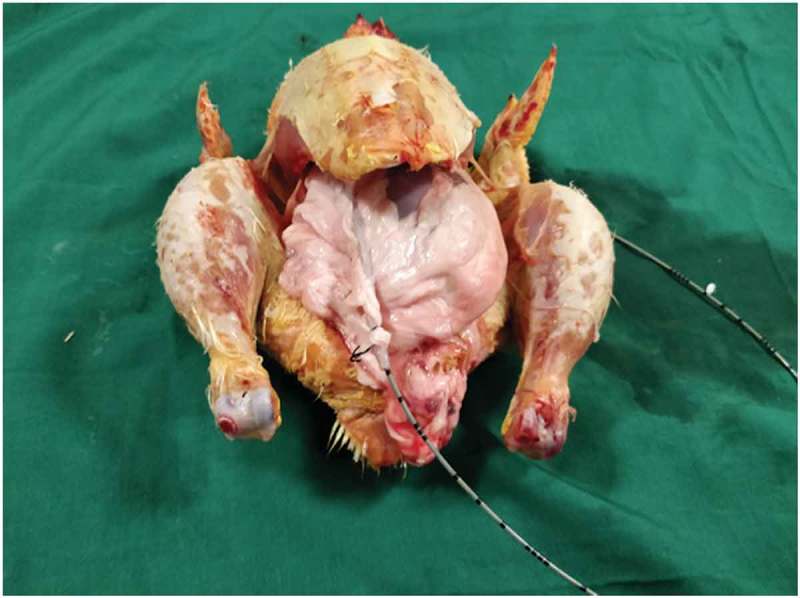
Bovine kidney placed inside chicken carcass.

**Figure 4.**
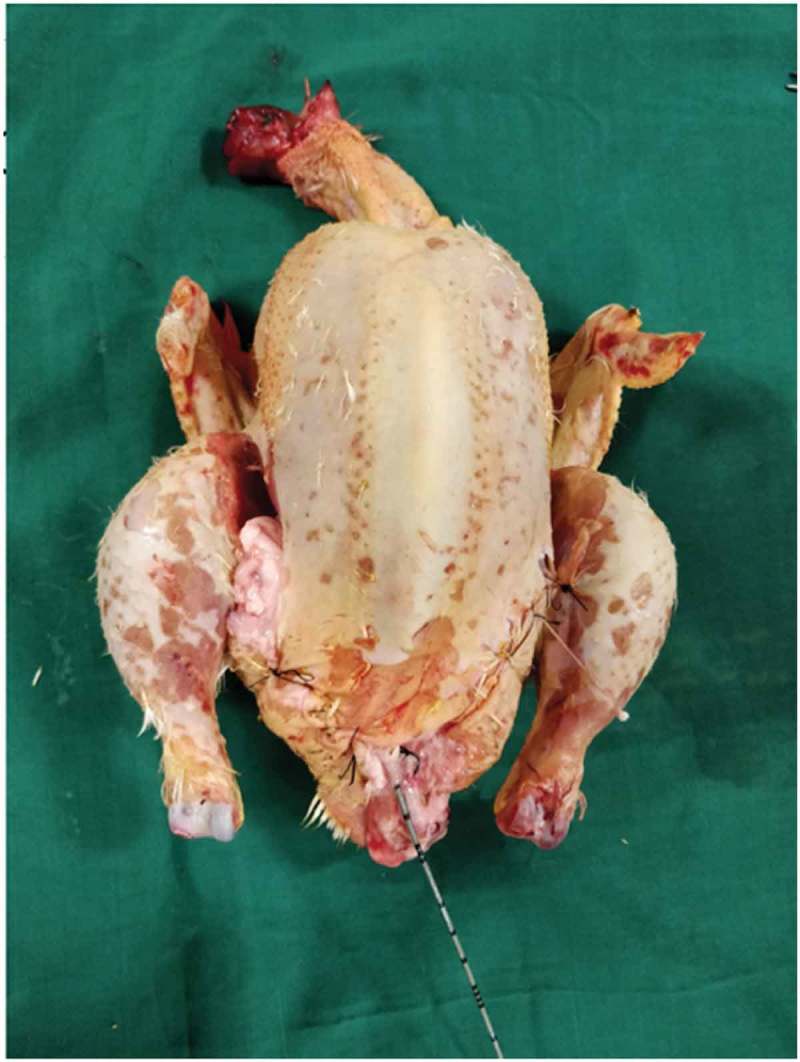
Final biological model with layers sutured.

Both the bovine kidney and the chicken were purchased from the slaughter house. Specific precautions were taken whilst procuring the bovine kidney so as to preserve maximal ureteric length and perinephric fat. The ureter is identified and a ureteric catheter of adequate size is placed in the ureter to the renal pelvis. The ureteric catheter is fixed to the ureter by a silk thread (Figure 1). The chicken carcass is completely eviscerated and cleaned so that adequate space is made for placement of the bovine kidney (Figure 2). The bovine kidney is placed in such a way that the calyces are perpendicular to the chicken (Figure 3). After placing the bovine kidney inside the chicken carcass and confirming the position, both the walls of the chicken carcass are sutured keeping the ureteric catheter outside (Figure 4).

### Content and construct validity

Content validity is the opinion of experts about the new *ex vivo* biological model [7]. This was done with the help of six senior urologists who had experience of ≥100 PCNLs. The experience in the expert group ranged between 320 and 5400 cases. They were asked to perform PRA in the new biological model and rate their experience on a 5-point Likert scale, which had four items.

Construct validity is the ability of the model to distinguish between different levels of experience between groups [7]. This was done by asking a group of 20 participants, which included 10 senior urologists (experts) with experience of >100 PCNLs and 10 junior urology residents (novices) with minimal experience of PCNLs. The novices had experience of ≤20 cases. They were asked to perform the PRA in the new biological model. The parameters assessed were time to puncture, attempts to successful puncture, fluoroscopy exposure time, and they also completed a subjective assessment questionnaire. Table 1 lists the equipment required.

**Table 1.** Equipment required.

Fluoroscopic machine Chicken carcass Bovine kidney Mannequin model 5-F ureteric catheter Contrast medium Glide wire Puncture needle

### Comparison with the mannequin model

**Figure 5.**
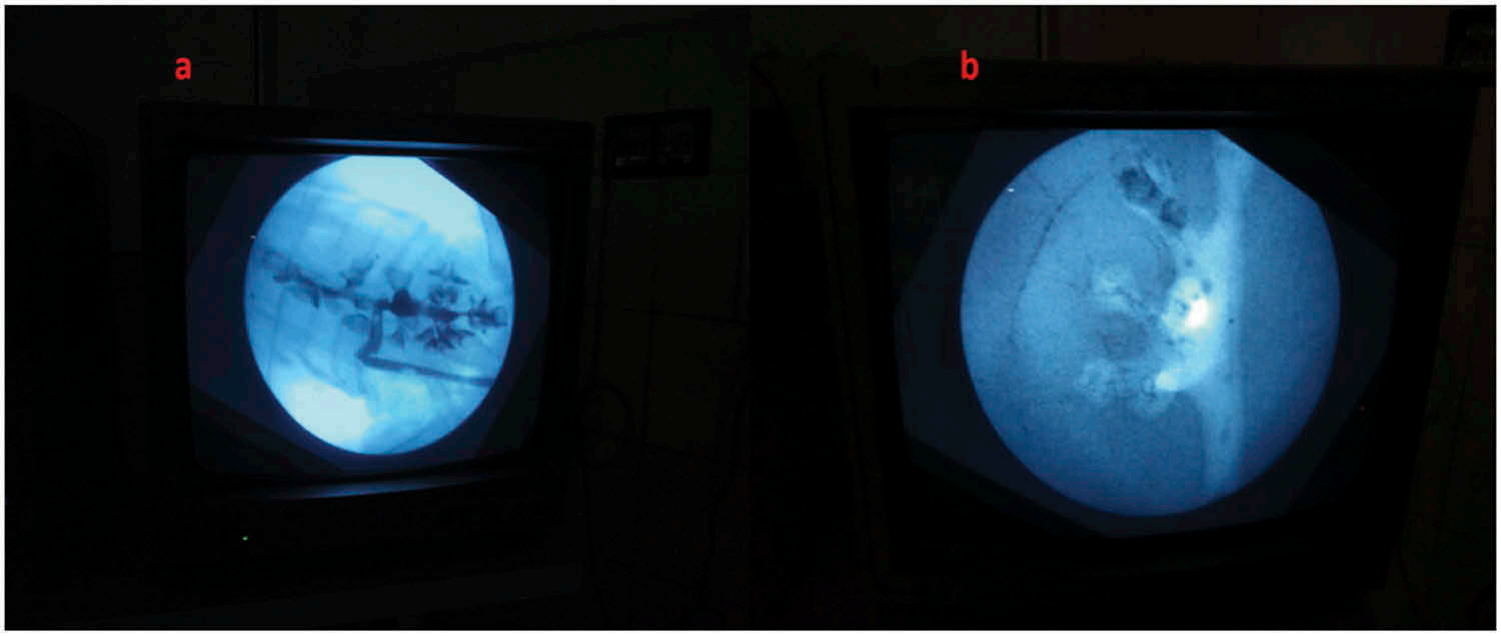
Fluoroscopic view of: (a) Biological model and (b) Non-biological model.

**Figure 6.**
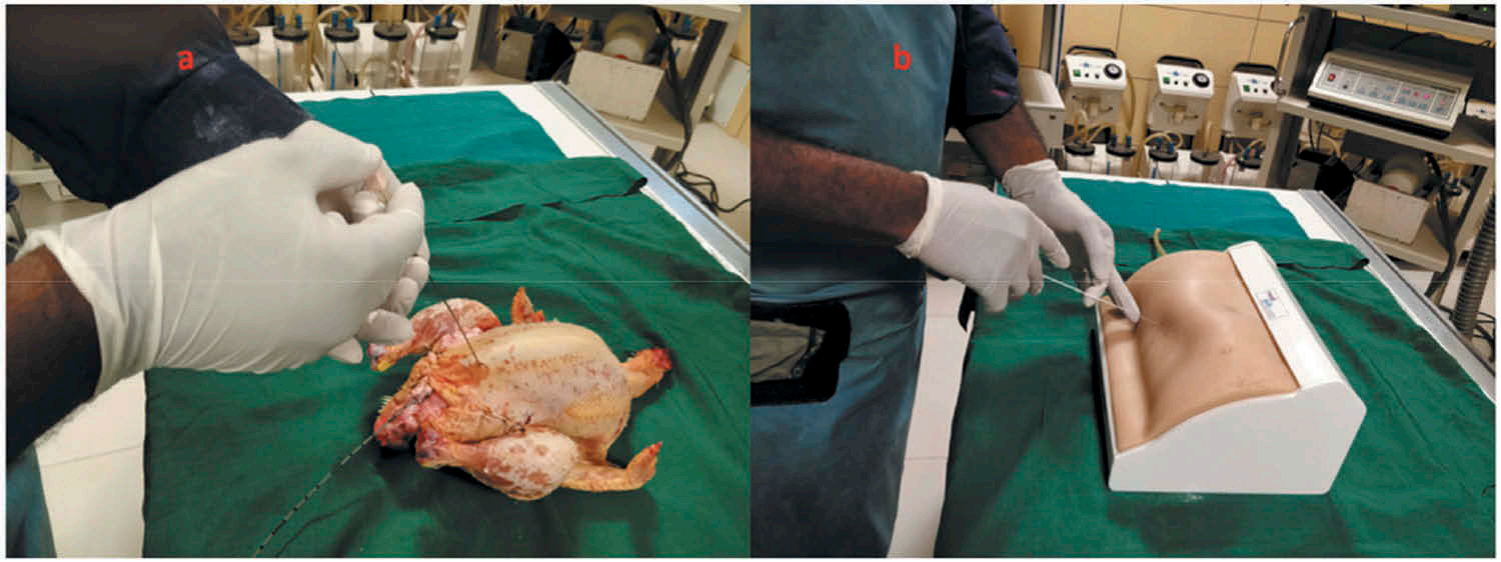
Surface view of puncture in: (a) Biological model and (b) Non-biological model.

**Figure 7.**
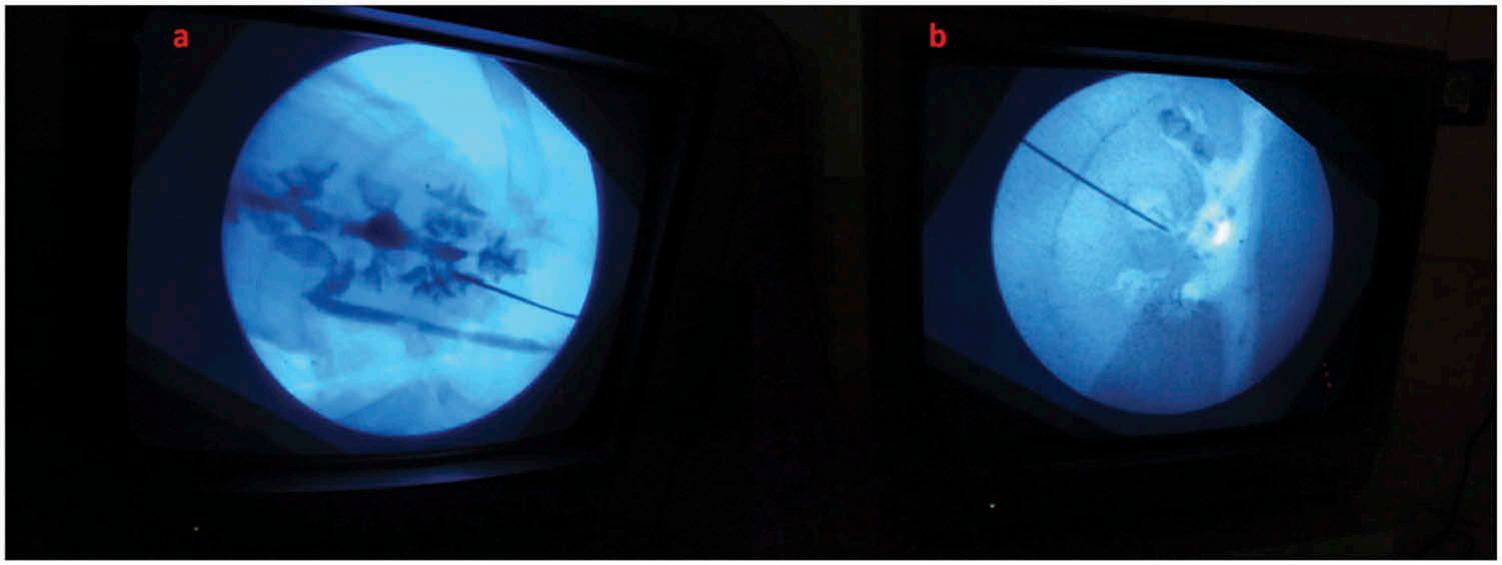
Fluoroscopic view of puncture in: (a) Biological model and (b) Non-biological model.

After the content and construct validity were achieved, the biological model was compared with the traditional non-biological model. In all, 20 urology residents with experience in PCNL ranging from five to 20 cases were enrolled. They were informed about the model and its function in detail by the author and were also helped by the author whilst performing the punctures. They were asked to do the punctures both in the biological and the non-biological model. The puncture technique was standardised and the puncture was done using the triangulation technique (Figure 5–7). This technique was followed as per institutional policy, as all the residents and consultants were trained and comfortable with the triangulation technique. The parameters assessed were time to puncture, attempts to successful puncture, and fluoroscopy exposure time. The participants also completed a subjective questionnaire on a Likert scale, which had six items relating to puncture. They were the ease of puncture, tissue feel, similarity to real PCNL, gaining confidence after training, and also about the preparation of the model.

Statistical analysis was carried out using the Statistical Package for the Social Sciences (SPSS®), version 15.0 (SPSS Inc., Chicago, IL, USA). The results were tabulated using the basic theory of statistics. The Student’s *t*-test was used for testing differences between means ± SDs, with *P* < 0.05 treated as statistically significant.

## Results

The new biological model was first tested for content and construct validity.

For the content validity, six senior urologists with experience of ≥100 PCNLs were briefed about the model and were asked to complete a Likert-scale questionnaire. The experts opined that the bovine kidney in the new biological model had good resemblance to the actual PCNL and was an excellent training tool. The results are summarised in Table 2.

**Table 2.** Content validity.

Number	Assessment field	Score (range 1–5)
1	Overall assessment	4
2	Ease of the model	3
3	Training tool	4
4	Resemblance with real-life scenario	5

For construct validity, 10 senior urologists (experts) with experience of ≥100 PCNLs were compared with 10 junior urology residents (novices) with very little PCNL experience. It was shown that the experts performed significantly better than the novices in all the parameters. The overall duration of the procedure was less for the experts compared to the novices, and their fluoroscopy exposure time was also significantly less. The experts were able to achieve successful puncture with fewer attempts compared to novices. The results are summarised in Table 3.

**Table 3.** Construct validity (experts and novices).

Variable, mean (SD)	Expert (*n* = 10)	Novices (*n* = 10)	*P*
Time to puncture, s	72.5 (18.27)	136.6 (42.29)	<0.001
Attempts to successful puncture, *n*	1.9 (0.73)	4.2 (0.91)	<0.001
Fluoroscopy exposure time, s	55.7 (16.73)	104.3 (45.56)	0.005

**Figure 8.**
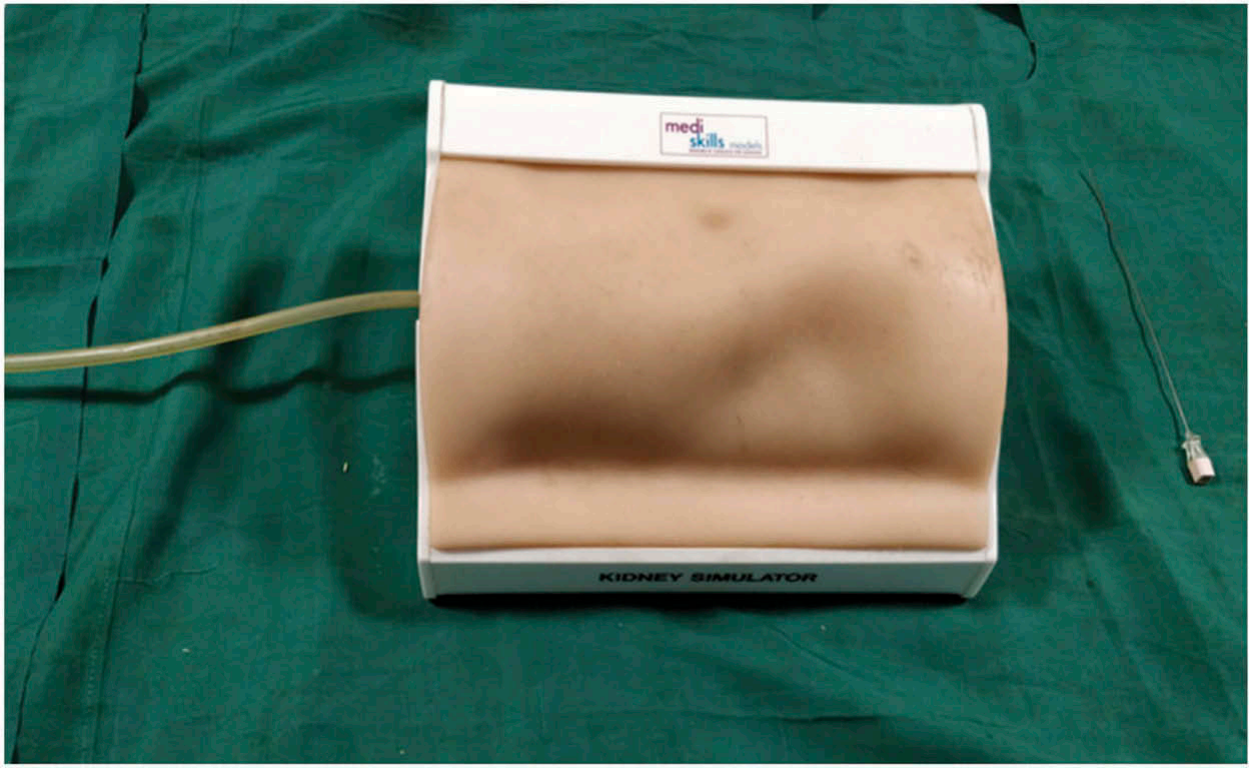
Non-biological model.

The new biological model was compared with the non-biological model (Figure 8). There was no statistically significant difference between the two models for time to puncture, total fluoroscopy exposure, and also the number of attempts taken for a successful puncture.

The participants were asked to rate the experience of both the training models by completing a subjective assessment questionnaire. This was a 5-point Likert scale with six items. The participants felt that the new biological model was better than the non-biological model for overall assessment and the tissue feel, with the biological model closely resembling the human tissue compared with the mannequin model. The participants also felt more confident after training in the biological model than the non-biological model. But the participants felt that the mannequin model was better than the bovine model when it came to ease of puncture and also the ease of preparation of the model. The results are summarised in Tables 4 and 5.

**Table 4.** Comparison between the new model and mannequin model: objective assessment.

Variable, mean (SD)	Biological model (*n* = 20)	Mannequin model (*n* = 20)	*P*
Time to puncture, s	92.7 (22.83)	88.4 (22.59)	0.553
Attempts to successful puncture, *n*	2.3 (0.92)	1.8 (0.83)	0.080
Fluoroscopy exposure time, s	74.75 (22.04)	67.85 (24).54	0.355

**Table 5.** Comparison between the new model and mannequin model: subjective assessment.

Assessment field score, mean (SD)	Biological model (*n* = 20)	Mannequin model (*n* = 20)	*P*
Overall assessment	4.4 (0.75)	2.95 (0.75)	<0.001
Ease of puncture	3.5 (0.88)	4.5 (0.68)	<0.001
Tissue feel	4.4 (0.75)	3.05 (0.68)	<0.001
Resemblance with real-life scenario	4.1 (0.78)	2.8 (0.61)	<0.001
Confidence after training	3.95 (0.68)	2.6 (0.59)	<0.001
Model preparation	2.7 (0.65)	4.8 (0.41)	<0.001

## Discussion

PCNL is a complex endourological procedure that requires a high level of expertise to perform. The most important and difficult to master step in PCNL is gaining PRA. Acquiring such expertise in a high tension environment, such as the operation theatre, can lead to steep learning curves and also increased cost. The use of simulators is essential to increase the quality of performance and reduce errors in real-life scenarios. The best example of this is the flight simulators used by pilots for training. These simulators have been found to be extremely helpful because the tasks in these models can be repeated any number of times and can be done in a controlled stress-free environment without the fear of complications in comparison to the real-life situation.

It has been proven that surgeons who practice on simulators tend to be more confident during the actual procedure and complete the procedure in less time with fewer complications.

The ideal training model should be able to mimic the actual procedure and should be inexpensive to construct. Our biological model was simple to construct and it resembled the actual procedure to a great extent because the bovine kidney pelvicalyceal system largely resembles the human kidney except that it has a small pelvis and multiple calyces. The multiple calyces were actually advantageous because it helped us in making more punctures in a single kidney; on average 15–20 punctures were made in a single bovine kidney.

Our biological model had content and construct validity, but the criterion validity could not be confirmed. A similar study conducted by Abdallah et al. [8] demonstrated the face validity of their biological model but did not demonstrate the content and construct validity. They used the bovine kidney in a human mannequin and compared different techniques of puncture. They demonstrated that the bovine kidney is feasible for learning PRA.

There is ample evidence in literature for biological and non-biological models for training in PRA.

The biological models either used porcine or bovine kidney alone or an entire animal. Hammond et al. [9] and Häcker et al. [10] used a porcine kidney in a chicken carcass. Zhang et al. [11], Qiu et al. [12] and Imkamp et al. [13] used porcine kidney encased in skin or tissue flaps. Earp [14] used porcine kidney encased in a foam layer. Kallidonis et al. [15] and Mishra et al. [16] studied the live anaesthetised pig as a model for PCNL training.

The non-biological models use either bench, mannequin or three-dimensional (3D) models, or VR simulators. The PERC Mentor^TM^ (Simbionix, Cleveland, OH, USA) is the only VR simulator validated for training in PRA [17,18]. Zhang et al. [19] used a novel kidney system made of silicone. Veneziano et al. [20] and Turney [21] advocated a novel 3D-printed model of the pelvicalyceal system for puncture.

After an extensive literature search, no evidence was found regarding use of a bovine kidney in a chicken carcass. Häcker et al. [10] have used a porcine kidney inside a chicken carcass and shown that an animal kidney could be easily placed inside a chicken carcass and could be used for training of PRA. However, they demonstrated ultrasound-guided access whereas in our present study we used fluoroscopy-guided access.

Even after an extensive literature search there was no evidence found regarding comparison of biological and non-biological models. Our present study demonstrated the content and construct validity of the model, which was found to be equally effective in training when compared with the non-biological model that had been used in our institute for training purposes. The biological model also scored better than the non-biological model in most of the subjective parameters assessed.

Our present model has some limitations. The bovine kidney anatomy is dissimilar to the human kidney with crowded calyces and a very narrow pelvis. This model only allows the practicing of the initial step of PCNL, i.e. the puncturing. The subsequent steps, such as tract dilatation and stone removal, cannot be practiced in this model. The movement of kidney and complications could not be reproduced. Even though the tissue feel is good compared to the mannequin model it is far inferior compared with the real-life scenario. This model was used only for fluoroscopy-guided puncture and not ultrasound-guided puncture. Additionally, the number of participants was limited.

## Conclusion

The present model was easy and inexpensive to construct, and has both content and construct validity. It is a feasible model for fluoroscopy-guided PRA. The bovine kidney in a chicken carcass model was equally effective when compared to the mannequin model for overall time and fluoroscopy exposure, and scored better than the mannequin model for tissue feel and the overall resemblance to human kidney puncture.
